# Identification of decreased intrinsic capacity: Performance of diagnostic measures of the ICOPE Screening tool in community dwelling older people in the VIMCI study

**DOI:** 10.1186/s12877-023-03799-0

**Published:** 2023-02-21

**Authors:** Xavier Rojano i Luque, Sergi Blancafort-Alias, Susanna Prat Casanovas, Susanna Forné, Nuria Martín Vergara, Pilar Fabregat Povill, Maria Vila Royo, Rosa Serrano, Dolores Sanchez-Rodriguez, Montserrat Vílchez Saldaña, Iris Martínez, Mariola Domínguez López, Francesc Riba Porquet, Aimar Intxaurrondo González, Antoni Salvà Casanovas

**Affiliations:** 1 Fundació Salut i Envelliment UAB (FSIE-UAB), Barcelona, Spain; 2 Sant Pau Institute for Biomedical Research (IIB Sant Pau), Barcelona, Spain; 3 ABS Ripoll-Sant Joan de les Abadesses, Institut Català de la Salut (ICS), Ripoll, Spain; 4 Fundació Sant Hospital, La Seu d’Urgell, Lleida, Spain; 5 EAP Horts de Miró, Institut Català de la Salut (ICS), Reus, Spain; 6 Hospital de la Santa Creu, Tortosa, Spain; 7 Badalona Serveis Assistencials (BSA), Badalona, Spain; 8 EDP Salut Sant Joan, Reus, Spain; 9 Department, Brugmann University Hospital, Université Libre de Bruxelles, Brussels, Belgium; 10 Health Organization Collaborating Center for Public Health Aspects of Musculo-Skeletal Health and Ageing, Division of Public Health, Epidemiology and Health Economics, University of Liège, Liège, Belgium; 11 Geriatrics Department, Rehabilitation Research Group, Hospital del Mar Medical Research Institute (IMIM), Barcelona, Spain; 12 ABS CamprodonInstitut Català de la Salut (ICS), Camprodon, Spain

**Keywords:** Intrinsic capacity, ICOPE, Screening, Sensitivity, Specificity, Person-centered care, Agreement, Diagnostic accuracy, Psychometrics

## Abstract

**Background:**

The World Health Organization (WHO) has developed the Integrated Care for Older People (ICOPE) strategy to face the challenges of ageing societies. This strategy is focused on person centered care and the assessment intrinsic capacity (IC). Early identification of five domains of IC (cognition, locomotion, vitality, sensory (hearing and vision), and psychological) has been shown to be related with adverse outcomes and can guide actions towards primary prevention and healthy ageing. IC assessment proposed by the WHO ICOPE guidelines is composed by two steps: First, Screening for decreased IC by the ICOPE Screening tool; second, by the reference standard methods. The aim was to assess the performance of diagnostic measures (sensibility, specificity, diagnostic accuracy, and agreement of the ICOPE Screening tool) compared to the reference standard methods in European community-dwelling older adults.

**Methods:**

Cross-sectional analysis of the baseline of the ongoing VIMCI (Validity of an Instrument to Measure Intrinsic Capacity) cohort study, which was carried out in Primary Care centers and outpatient clinics from 5 rural and urban territories in Catalonia (Spain). Participants were 207community dwelling persons ≥ 70-year-old with Barthel $\ge$ 90, without dementia or advanced chronic conditions who provided their consent to participate. The 5 IC domains were assessed by the ICOPE Screening tool and the reference methods (SPPB, gait speed, MNA, Snellen chart, audiometry, MMSE, GDS5) during patients’ visit. Agreement was assessed with the Gwet AC1 index.

**Results:**

ICOPE Screening tool sensitivity was higher for cognition (0.889) and ranged between 0.438 and 0.569 for most domains. Specificity ranged from 0.682 to 0.96, diagnostic accuracy from 0.627 to 0.879, Youden index from 0.12 to 0.619, and Gwet AC1 from 0.275 to 0.842.

**Conclusion:**

The ICOPE screening tool showed fair performance of diagnostic measures; it was helpful to identify those participants with satisfactory IC and showed a modest ability to identify decreased IC in older people with high degree of autonomy. Since low sensitivities were found, a process of external validation would be recommended to reach better discrimination. Further studies about the ICOPE Screening tool and its performance of diagnostic measures in different populations are urgently required.

## Background

The World Health Organization (WHO) launched the World Report on Ageing and Health in 2016 [1, 2], which recommended major systemic changes in the health systems in order to adopt person-centered care models which enable “healthy ageing”. Healthy ageing [1] is the overall goal of the Global Strategy and Action Plan on Ageing and Health [3] and the current Decade of Healthy Ageing plan developed by the WHO [4] and is defined as “the process of developing and maintaining the functional ability that enables well-being in older age”. Functional ability is determined by three factors: Intrinsic capacity (IC), which is “the composite of all the physical and mental capacities of an individual”, the environment, and the interactions between both. IC is composed by five domains: Cognition, locomotion, vitality (related to energy balance and metabolism), sensory (related to vision and audition), and psychological (related to mood and depressive symptoms) [5]. Declines in IC of any of its domains have been shown to be related to adverse health outcomes [6]; Importantly, decreased IC is reversible and can be counteracted when early identification and treatment are applied [7].

For this reason, the WHO has fostered the Integrated Care for Older People (ICOPE) strategy [8], the WHO ICOPE Guidelines on Community-Level Interventions to Manage Declines in Intrinsic Capacity[7], and the WHO ICOPE Guidance for person-centered assessment and pathways in Primary Care[9], aimed at the preservation of IC in community-dwelling older populations. The WHO ICOPE strategy is based on five consecutive steps: 1) Screen for declines in IC; 2) Undertake a person-centered assessment in Primary Care; 3) Define care goals and tailor a personalized care plan; 4) Ensure a referral pathway and care plan monitoring, with links to specialized geriatric care; 5) Engage communities and support caregivers.

In order to carry out the first step, the WHO has developed the ICOPE Screening tool [9], an evidence-based 9-item questionnaire which uses the principles of comprehensive geriatric assessment to identify deficiencies in the 5 IC domains., and which is recommended in the WHO ICOPE guidelines [7, 9].

The ICOPE strategy has been described as potentially beneficial in a primary care context for several reasons [9]. First, as a simple and low-cost way to identify decreased IC in older people and provide appropriate care to reverse or slow down the decline. Secondly, as a support for the inclusion of services to prevent care-dependency and the creation of a partnership involving older people, primary health-care professionals, family, and community. Thirdly, ICOPE interventions are designed to be provided through models of care that prioritize primary and community-based care. The ICOPE Screening tool is in process of being tested as a pilot instrument in few countries [10, 11]. Preliminary findings highlight its potential as an inexpensive, feasible tool, easy to be administered in settings with limited resources, which requires no specific training, and is extremely time-efficient. However, the ICOPE Screening tool is relatively new, and the evidence about its performance of diagnostic measures still remains unavailable; gathering information about them is crucial, since it provides data that will help proceed towards the implementation of the the first step of the ICOPE strategy.

The objective was to assess the performance of diagnostic measures (sensibility, specificity, diagnostic accuracy, and agreement) of the ICOPE Screening tool, recommended by the WHO ICOPE guidelines, to detect decreased IC, compared to the reference standard methods, in European community-dwelling older adults from the VIMCI (Validity of an Instrument to Measure Intrinsic Capacity) cohort study.

## Methods

### Settings

Cross-sectional analysis of the baseline data of the VIMCI study, a cohort study aimed at assessing the performance of diagnostic measures of the ICOPE Screening tool and the changes in intrinsic capacity during a 12-month follow-up. Baseline assessment was conducted between April and September 2021. The study included community-dwelling older people aged 70 or over with a Barthel index ≥ 90, recruited in Primary Care centers and outpatient clinics from five rural and urban territories in Catalonia (Spain). Participants with dementia, advanced chronic conditions, or a life expectancy < 12 months were excluded. The VIMCI study is part of the larger European APTITUDE project[12], aimed at preventing the dependency on older people by creating a network to promote care, training, research, and innovation in the areas of public health and gerontology.

### Sampling

An intended convenience sample of 250 participants was determined for the study, and 207 were finally included. The use of a convenience sample was determined by budgetary constraints, the requirement to recruit participants within a limited timeframe and the fact that we didn’t have access to the full target population to create a representative sample.

### IC assessment

IC assessment according to the WHO ICOPE guidelines comprised two steps: First, screening by the ICOPE Screening tool and second, to undertake a complete person-centered assessment by the reference standard methods. In the VIMCI study, the ICOPE Screening tool was administered before the reference standard methods during the same visit. The whole assessment of each participant was performed by one single person. Participants using sensory or mobility aids (i.e., glasses and canes) were allowed to use them during the assessments to ensure participants’ safety. Table 1 summarizes the IC assessment by the ICOPE Screening tool and the reference standard methods in the VIMCI study.

**Table 1:** IC loss criteria

Domain	ICOPE	Reference
Cognition	- Any time or space orientation failure OR—not recalling 3 words	MEC score < 24
Locomotion	Spend > 14 s in 5 times chair rise	- SPPB score < 10 OR—gait speed ≤ 0.8 m/s
Vitality	- Unintentional loss of > 3 kg over the last 3 months OR—Appetite loss	MNA score < 12
Sensory—Vision	Reporting sight problems	visual acuity < 6/60 in tumbling E chart
Sensory—Hearing	Failing to repeat a minimum of 4 words spelled in a whisper voice with at least one ear	light hearing loss in one ear (Hearing test audiogram score < 6)
Psychological	Being bothered over the past two weeks – feeling down, depressed or hopeless OR – little interest or pleasure in doing things	GDS5 ≥ 2

*MEC* Mini Cognitive Examination, *SPPB* Short Physical Performance Battery, *MNA* Mini Nutritional Assessment, *GDS5* 5-item version of the Geriatric Depression Scale

#### IC Screening by the ICOPE Screening tool

The screening of decreased IC was performed by the ICOPE Screening tool, following the standardized procedures detailed in the WHO ICOPE guideline [13]. The ICOPE Screening tool is a 9-item instrument which assesses the domains of cognition (2 items), mobility (1 item), vitality (2 items), sensorial (1 item for vision and 1 item for audition), and psychological capacity (2 items). The result of the ICOPE Screening tool classifies each IC domain as normal or altered. An altered domain indicates that a further assessment with the reference standard method is required; for purpose of analysis, both the screening and the reference standard method were administered to all study sample, independently of the result obtained in the screening. In presence of two options to assess each of the domains during the screening, only one of the two was chosen for each participant. In the case of audition, the whisper test, which does not need additional equipment, was the only proposed option.

#### IC assessment by the reference standard methods

For each IC domain, the reference standard methods were administered according to the recommendations of the WHO ICOPE guideline [13]. For each participant, each domain was considered normal or altered according to the thresholds for the initial validation for each reference standard method.

- Cognitive domain: Mini Cognitive Examination (MEC), which is a Spanish adaptation of the Mini Mental State Examination (MMSE) was used and a MEC score < 24 indicated an altered domain [14, 15].
- Locomotion domain: Short Physical Performance Battery (SPPB) [16], a 3-step composite test that assesses balance, gait speed, and lower limb strength. Both SPPB total score and gait speed alone and were used as reference tests for this domain. A SPPB score < 10 or a gait speed ≤ 0.8 m/s, respectively, were used as thresholds[17, 18].
- Vitality domain: The Mini Nutritional Assessment (MNA) by using a threshold MNA score < 12 was used [19].
- Sensorial domain—Audition: The Hearing test Audiogram App [20]using a wired headphone connected to a smartphone was used. The App uses pure tones at 125, 250, 500 Hz and 1, 2, 3, 4 and 8 kHz. The App creates an audiogram and provides a score between 1 (very severe hearing loss) to 8 (hearing better than average). To assess frequency of sensory problems the data of hearing and vision tests was combined, considering the domain normal when both tests were negative; altered when one met decreased IC criteria; and missing when one was normal and the other had no data.
- Psychological domain: The 5-item version of the Geriatric Depression Scale (GDS5) was administered and a threshold of GDS5 ≥ 2 was used [21].
- Demographic, social, and health variables were gathered. Level of education was determined according to self-declared level of finished studies. Risk of social isolation was classified according to the Lubben Social Network Scale–Revised (LSNS-R)) [22] as low (31—60 points), moderate (26—30), high (21—25), and isolated (0—20). Loneliness was assessed by De Jong Gierveld Loneliness Scale (JGLS) [23] considering four levels: absent (0 – 2 points), moderate (3 – 8), severe (9 – 10), and very severe (11 points). Self-perceived health and amount of health problems were self-reported. Severity of comorbidities were assessed with the Charlson index[24].

### Outcome measure

The outcome measure was the performance of diagnostic measures of the ICOPE Screening tool compared to the reference standard methods for each domain: Sensibility, specificity, diagnostic accuracy, and the Youden index [25]. A screening tool is considered to have good performance indicators, if sensitivity and specificity > 80%; fair, if sensitivity or specificity < 80% but both values > 50%; and poor, if sensitivity or specificity < 50% [26].

The Youden index summarizes sensibility and specificity, and it is frequently used with dichotomous variables instead of Receiver Operating Characteristic (ROC) curves. A Youden index of 1 indicates perfect sensibility and specificity, and a value of 0 indicates that the test is not useful at all.

Agreement between the ICOPE Screening tool and the reference standard methods was assessed using the GwetAC1 index [27], a chance corrected indicator analogous to Cohen’s kappa but more robust to sample distribution[28]. Higher values indicate better agreement (usually 0.4 to 0.6 are considered moderate, 0.6 to 0.8 good, and 0.8 to 1 very good). In the case of the mobility domain, the performance of diagnostic measures and agreement were calculated for both SPPB and gait speed. For the audition domain, additional analysis considering the results of the audiogram in the best ear was conducted.

### Statistics

Quantitative variables were analyzed using the Mann Whitney U test. Qualitative variables were analyzed using Pearson’s Chi-squared test or Fisher’s exact test. All participants underwent the ICOPE assessment, but some subjects had missing reference tests. In those cases, subjects with missing information for a reference test were excluded for the analysis of agreement and diagnostic measures of the affected domain but were used for the analysis of the other domains for which data was available. Statistical analysis was performed using R 4.2.1 and epiR 2.0.52 and irrCAC 1.0 libraries.

## Results

Two hundred seven participants (61% women) were included in the study and their characteristics are shown in Table 2 and 3. Men were affected by more severe conditions than women, although no differences were found in health state perception (rated positively in 66% of cases) nor the amount of health issues. Nearly one in four participants (24%) reported some degree of isolation and more than a third (37%) felt lonely.

**Table 2:** Characteristics of the participants

Characteristic	Overall, *N* = 207	Men, *N* = 81	Women, *N* = 126	*p*-value
Age	76.7 (74.1 to 79.9)	77.4 (74.8 to 80.4)	76.3 (73.3 to 79.4)	0.072
Level of education				
No studies	30 (14%)	12 (15%)	18 (14%)	0.598
Primary	107 (52%)	38 (47%)	69 (55%)	
Secondary	46 (22%)	19 (23%)	27 (21%)	
University	24 (12%)	12 (15%)	12 (9.5%)	
Perceived health				
Very good	31 (16%)	12 (16%)	19 (15%)	0.747
Good	100 (50%)	41 (54%)	59 (48%)	
Fair	51 (26%)	18 (24%)	33 (27%)	
Bad	13 (6.5%)	3 (3.9%)	10 (8.1%)	
Very bad	4 (2.0%)	2 (2.6%)	2 (1.6%)	
Unknown	8	5	3	
Number of health problems	4.00 (2.00 to 5.00)	3.00 (2.00 to 5.00)	4.00 (2.00 to 6.00)	0.246
Charlson index	1.00 (0.00 to 2.00)	1.00 (0.00 to 2.00)	1.00 (0.00 to 1.00)	0.008
LSNS score	38 (31 to 45)	38 (31 to 44)	38 (31 to 45)	0.838
Unknown	6	4	2	
Social isolation risk				
Low	153 (76%)	58 (75%)	95 (77%)	0.960
Moderate	23 (11%)	10 (13%)	13 (10%)	
High	17 (8.5%)	6 (7.8%)	11 (8.9%)	
Isolated	8 (4.0%)	3 (3.9%)	5 (4.0%)	
Unknown	6	4	2	
JGLS score	2.00 (0.00 to 3.00)	2.00 (0.00 to 3.00)	2.00 (0.00 to 4.00)	0.723
Unknown	6	4	2	
Degree of loneliness				
Does not have any	127 (63%)	48 (62%)	79 (64%)	0.935
Moderate	70 (35%)	28 (36%)	42 (34%)	
Severe	3 (1.5%)	1 (1.3%)	2 (1.6%)	
Very severe	1 (0.5%)	0 (0%)	1 (0.8%)	
Unknown	6	4	2	

Continuous values expressed as median an interquartile rank. *LSNS* Lubben Social Network Scale, *JGLS* De Jong Gierveld loneliness scale

**Table 3:** Frequency of decreased IC according to screening and reference criteria

Domain	ICOPE^a^	Reference^a^
Sensory	112/207 (54%)	120/204 (59%)
Hearing	67/207 (32%)	104/204 (51%)
Vision	70/207 (34%)	32/205 (16%)
Locomotion	57/207 (28%)	107/202 (53%)
Psychological	49/207 (24%)	34/204 (17%)
Vitality	22/207 (11%)	33/206 (16%)
Cognition	64/207 (31%)	9/202 (4.5%)

^a^cases/number of observations (valid percent)

**Fig. 1:**
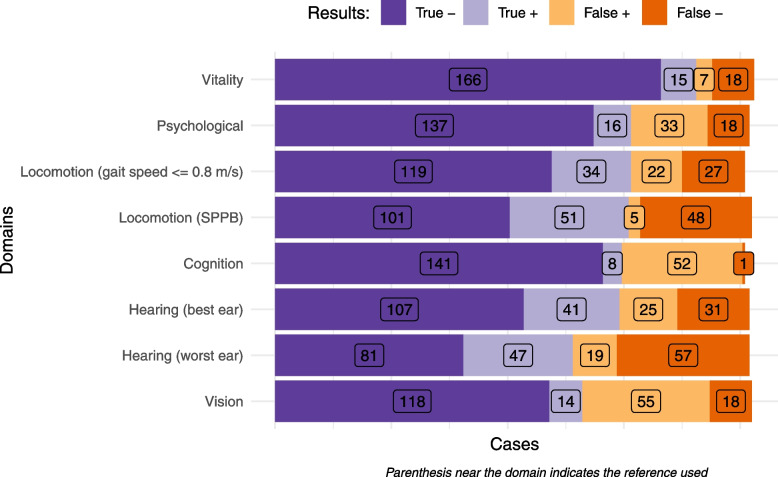
Results of assessments for each domain

Figure 1 shows for each domain the distribution of participants in four categories according to the results of screening and reference tests ( Domains are sorted by their average diagnostic accuracy. Purple bars on the left show participants classified in the same way: dark purple show true negative cases (both tests discard the condition), and light purple true positive (both tests detect the condition). Orange bars on the right show discordant cases: light orange show false positive cases (the condition is detected in the screening, but not by the refence test), and dark orange show false negative cases (the screening test fails to detect the condition).

Frequency of decreased IC is shown in Table 4 and 5 (sort in descending order according to reference method). Seventy percent and 79% of participants showed at least one domain with decreased IC according to the ICOPE Screening tool and the reference standard methods, respectively. These percentages dropped to 52% and 60% if sensory domain was not considered. Among participants with decreased IC in least one domain, the mean number of domains affected were 2.1 using ICOPE Screening tool and 1.8 using the reference standard methods (1.8 and 1.4 excluding the sensory domain).

**Table 4:**
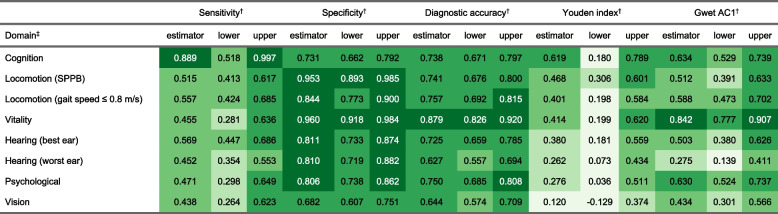
Psychometric and agreement indicators

†Point estimator with upper and lower limit of 95% confidence interval; ‡Parenthesis indicates reference used (when several)

**Table 5:** Reference tests scores

	Screening result		
Reference	Normal	Altered	*p*-value
MEC score	*n* = 141; 29.00 (28.00 to 30.00)	*n* = 52; 28.00 (25.97 to 28.00)	< 0.001
MEC altered (0 to 23)	1 (0.7%)	8 (13%)	< 0.001
SPPB score	*n* = 149; 10.00 (9.00 to 11.00)	*n* = 56; 7.00 (6.75 to 8.25)	< 0.001
SPPB category			
0 to 3 points	0 (0%)	5 (8.9%)	< 0.001
4 to 6 points	7 (4.7%)	9 (16%)	
7 to 9 points	41 (28%)	37 (66%)	
10 to 12 points	101 (68%)	5 (8.9%)	
Maximum speed (m / s)	*n* = 144; 1.01 (0.86 to 1.17)	*n* = 54; 0.68 (0.57 to 0.91)	< 0.001
Slowness (≤ 0.8 m/s)	27 (18%)	34 (61%)	< 0.001
MNA-SF: score	*n* = 184; 14.00 (13.00 to 14.00)	*n* = 22; 10.00 (9.25 to 12.75)	< 0.001
MNA-SF: category			
Malnutrition	2 (1.1%)	0 (0%)	< 0.001
Risk of malnutrition	16 (8.7%)	15 (68%)	
Normal	166 (90%)	7 (32%)	
GDS5: symptoms	*n* = 155; 1.00 (1.00 to 1.00)	*n* = 49; 1.00 (1.00 to 2.00)	0.002
GDS5: presence of depressive symptoms	18 (12%)	16 (33%)	0.002
Visual acuity			
Normal vision (≥ 6/18)	118 (87%)	55 (80%)	0.055
Slight vision reduction (6/60)	16 (12%)	11 (16%)	
Moderate vision reduction (3/60)	0 (0%)	3 (4.3%)	
Severe vision impairment (< 3/60)	2 (1.5%)	0 (0%)	
Best ear hearing			
Better than average	1 (0.7%)	0 (0%)	< 0.001
Normal hearing	73 (53%)	10 (15%)	
Minimal hearing loss	33 (24%)	15 (23%)	
Low hearing loss	17 (12%)	17 (26%)	
Moderate hearing loss	10 (7.2%)	15 (23%)	
Moderate-severe hearing loss	4 (2.9%)	7 (11%)	
Severe hearing loss	0 (0%)	2 (3.0%)	
Worst ear hearing			
Normal hearing	53 (38%)	6 (9.1%)	< 0.001
Minimal hearing loss	28 (20%)	13 (20%)	
Low hearing loss	32 (23%)	12 (18%)	
Moderate hearing loss	12 (8.7%)	18 (27%)	
Moderate-severe hearing loss	9 (6.5%)	12 (18%)	
Severe hearing loss	4 (2.9%)	5 (7.6%)	

For continuous variables valid cases, median and interquartile rank. For categorical variables valid cases and valid column percent

Sensibility, specificity, diagnostic accuracy, Youden and Gwet AC1 indexes are shown in Table 2 and 3. The domains are sorted by Youden’s index. Sensitivity ranged for most domains between 0.438 and 0.569 being higher for cognition (0.889). Specificity ranged from 0.682 to 0.96, diagnostic accuracy from 0.627 to 0.879, Youden index from 0.12 to 0.619, and Gwet AC1 from 0.275 to 0.842.

**Fig.2:**
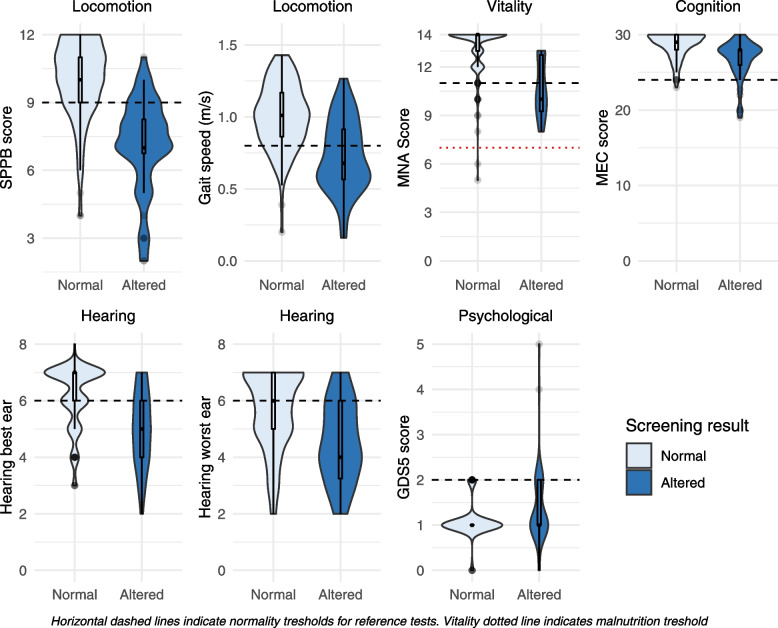
Reference scores according to screening result

Figure 2 shows the boxplot and density distribution of reference tests -on the vertical axis- according to the results of the ICOPE Screening test. For all domains but psychological (where a higher score indicates higher risk), the area over the dashed line shows the proportion of participants with normal scores. Table 3 and 4 shows reference results for each domain according to the results of its corresponding ICOPE Screening tool. From the 207 participants, 9 (4%) had an altered MEC score. Nearly half of participants (48%) showed decreased mobility according to SPPB score, mostly with mild severity (78 out 99). Thirty-three participants had a MNA below 12 points. In relation to sensory domain, 16% of participants were affected by vision impairment, mostly slight (84%, 27 out 32); and seven in 10 participants reported hearing problems, mostly with minimal or low loss. In all domains, the score in the reference standard methods was better in the group with normal results in the screening. Mean differences (95% confidence interval) were: 1.8 points (1.1 to 2.5) for MEC score; 2.9 (2.3 to 3.5) for SPPB; 0.3 m/s (0.2 to 0.4) for gait speed; 2.5 points (1.7 to 3.3) for MNA; and 0.3 (0.1 to 0.6) symptoms for GDS5.

## Discussion

The study assessed the performance of diagnostic measures (sensibility, specificity, and diagnostic accuracy) of the ICOPE Screening tool compared to the reference standard method for each IC domain in European community-dwelling older people participating in the VIMCI study. The ICOPE Screening tool showed to have fair performance of diagnostic measures, with a modest sensitivity and a good specificity for the screening of decreased IC among this sample of community-dwelling older people. Diagnostic accuracy was found fair for all domains. Youden’s index followed a gradient showing a poor performance for psychological, visual and hearing domains of IC. These relatively modest results in the ICOPE Screening tool could be explained by the eligibility criteria and characteristics of the study sample, which was composed by community dwelling older adults at high level of autonomy, and in a very early stage of the ageing process.

Several considerations about the findings in the five IC domains are worth discussing. The screening for the cognitive screening achieved the highest sensitivity. In the cognitive domain, participants with abnormal results in the ICOPE Screening tool obtained a lower score than those with normal results in the screening; even when such range of difference for the MEC has been shown to be clinically meaningful for people without cognitive impairment [29], the average score of both groups was within the range of normality [30]. For mobility, both SPPB score and gait speed also showed fair performance of diagnostic measures, as well as clinically meaningful differences between those with screening normal and abnormals [31], with averages below the normal threshold in the group with altered results in the screening. In the vitality domain, those with abnormal results in the screening obtained worse results in the MNA, with average scores below the normal threshold. In the psychological domain, the GDS5 showed worse results in those participants with a positive screening, although differfences were not found in the median score (between the normal range in both groups). Regarding the sensory domain, no differences between groups were found in vision. Finally, sensitivity results for audition seemed to be counter-intuitive, as a higher probability of detecting a hearing problem would be expected as its severity increases. Nevertheless, sensitivity was worse when we consider the worst ear. In relation to Gwet AC1, all non-sensory domains show moderate to good agreement with the reference standard methods, which may be explained because some items of the ICOPE Screening tests are based in constructs that are similarto the original reference standard method. To our knowledgthis is the first study comparing the results of the ICOPE screening with a reference test for each single dimension. A previous Chinese ICOPE pilot [11] compared results of gait speed, MMSE, and GDS30 score between persons with decreased IC in at least one domain. In this case those with all domains preserved found worse results in MMSE and GDS30in persons with decreased IC and no differences in gait speed. Nevertheless their analysis have a risk of classification bias that may underestimate the effects of decreased IC. Few studies [11, 32, 33] have published findings about the frequency of decreased IC, but substantial differences in inclusion criteria prevents a straightforward comparison with them.

Two limitations should be acknowledged for the study. First, there is a selection bias due to the healthy status of the study participants, with a sample not fully representative of the overall health status of older people aged 70 and over due to participation criteria and sampling methods. As a consequence, prevalence and all indicators relaying on it must be interpreted with caution and may hinder its generalization to general older population. Second, the IC sensory domain was assessed by a reference method that may have lower sensitivity and specificity than others considered as the “gold standard” [34] but the evidence about studies which include this domain among the IC construct is still scarce [35].

Four strengths of the study should be highlighted. First, the highest methodological quality in a prospective design with study procedures according to the ICOPE guidelines, developed specifically to test the current hypothesis were followed, which differs from most analysis about ICOPE, mostly post-hoc analysis and retrospective assessments. Second, the high quality of the information used, with a complete IC assessment for the 5 domains with very little missing data. Indeed, this is one of the few studies which include the sensory domain, measured by the appropriate instruments to detect sensory issues in a validated, feasible, and inexpensive way. This is extremely innovative, as information about the sensory, audition IC domain is frequently missing, because it may not be accessible in Primary Care health records, and if available is usually retrospectively collected. Third, one of the strongest points of this paper is its novelty and originality, as to authors’ knowledge, no evidence is available about the performance of diagnostic measures of the ICOPE Screening tool in their intended target population, which make the findings valuable to advance forward in the implementation of the ICOPE strategy. Finally, the study showed the innovative experience of a larger systematic program aimed at identifying IC deficits and implementing the WHO ICOPE approach as part of the decision-making process in clinical practice in a population level.

The detection of decreased IC in primary care is important because it has been associated to an increased risk of death, incident disability and dependence (independently of health problems), up to a 5-year follow-up[6, 36, 37]. A failure to recall 2 words or the day of the week has been shown to increase the risk of incident dementia[38], and a prolonged time performing the 5 times sit-to-stand test is associated with disability[39].

## Conclusions

The WHO ICOPE Screening tool showed fair performance indicators in healthy older community-dwelling population in initial stages of the ageing process in the VIMCI study. Despite the relatively limited results in the performance of diagnostic measures in this population, the ICOPE Screening tool highlighted as a simple, inexpensive, and feasible tool, and may be suitable to face the barriers of the implementation of the ICOPE strategy. Repeated assessments (i.e., every six months) in those people without abnormalities in the ICOPE screening tool may be a potential way to overcome the underestimation of decreased IC in those participants with normal initial results in the screening.

Nevertherless, further studies in different settings are required in order to assess the usefulness of the ICOPE screening tool and to establish proper recommendations.

## Data Availability

The datasets analysed during the current study are not publicly available because the informed consent stated that participants data will not be shared with any third party not related with the study. Data may be available from the corresponding author on reasonable request after the approval of the Ethics Committee and the Data Protection Delegate of the Universitat Autònoma de Barcelona.

## Abbreviations

GDS5: 5 items Geriatric Depression Scale
IC: Intrinsic Capacity
ICOPE: Integrated Care for Older People
IST: Icope Screening Tool
JGLS: de Jong Gierveld Loneliness Scale
LSNS-R: Lubben Social Network Scale–Revised
MEC: Mini Cognitive Examination
MNA: Mini Nutritional Assessment
SPPB: Short Physical Performance Battery
VIMCI: Validity of an Instrument to Measure Intrinsic Capacity
WHO: World Health Organization
